# The influence of urban trees and total vegetation on asthma development in children

**DOI:** 10.1097/EE9.0000000000000280

**Published:** 2023-11-16

**Authors:** Louise Duquesne, Elhadji Anassour Laouan Sidi, Céline Plante, Ying Liu, Naizhuo Zhao, Éric Lavigne, Kate Zinszer, Rita Sousa-Silva, Michel Fournier, Paul J. Villeneuve, David J. Kaiser, Audrey Smargiassi

**Affiliations:** a Department of Environmental and Occupational Health, School of Public Health, University of Montreal, Montreal, Canada; b National Institute of Public Health of Quebec, Montreal, Quebec, Canada; c Center for Public Health Research (CReSP), University of Montreal and CIUSSS du Centre-Sud-de-l’Île-de-Montréal, Montreal, Canada; d Centre for Forest Research, Université du Québec à Montréal, Montreal, Quebec, Canada; e Division of Clinical Epidemiology, McGill University Health Centre, Montreal, Quebec, Canada; f School of Epidemiology and Public Health, University of Ottawa, Ottawa, Ontario, Canada; g Water and Air Quality Bureau, Health Canada, Ottawa, Ontario, Canada; h Young Academy for Sustainability Research, Freiburg Institute for Advanced Studies, University of Freiburg, Germany; i Montreal Regional Department of Public health, CIUSSS du Centre-Sud-de-l’Île-de-Montréal, Montreal, Canada; j School of Mathematics and Statistics, Carleton University, Ottawa, Ontario, Canada; k Department of Epidemiology, Biostatistics and Occupational Health, McGill University, Montreal, Quebec, Canada

**Keywords:** asthma, child, cohort study, environmental health/epidemiology, pollen, trees, urban vegetation

## Abstract

**Objective:**

We aimed to assess whether the influence of urban vegetation on asthma development in children (<13 years) varies by type (e.g., total vegetation, tree type, and grass) and season.

**Methods:**

We used a cohort of all children born in Montreal, Canada, between 2000 and 2015. Children and cases were identified from linked medico-administrative databases. Exposure to residential vegetation was estimated using the Normalized Difference Vegetation Index (NDVI) for total vegetation and using the total area covered by deciduous and evergreen crowns for trees in 250 m buffers centered on residential postal codes. Seasonal variations in vegetation were modeled by setting values to zero on days outside of pollen and leaf-on seasons. Cox models with vegetation exposures, age as a time axis, and adjusted for sex, material deprivation, and health region were used to estimate hazard ratios (HR) for asthma development.

**Results:**

We followed 352,946 children for a total of 1,732,064 person-years and identified 30,816 incident cases of asthma. While annual vegetation (total and trees) measures did not appear to be associated with asthma development, models for pollen and leaf-on seasons yielded significant nonlinear associations. The risk of developing asthma was lower in children exposed to high levels (>33,300 m^2^) of deciduous crown area for the leaf-on season (HR = 0.69; 95% confidence interval [CI] = 0.67, 0.72) and increased for the pollen season (HR = 1.07; 95% CI =1.02, 1.12), compared with unexposed children. Similar results were found with the Normalized Difference Vegetation Index.

**Conclusion:**

The relationship between urban vegetation and childhood asthma development is nonlinear and influenced by vegetation characteristics, from protective during the leaf-on season to harmful during the pollen season.

**What this study adds**

Studies on vegetation exposure and asthma incidence yielded mixed results. Few provided insight into the influence of the components of vegetation and seasons on the association, as most used yearly measures of overall greenness, such as the Normalized Difference Vegetation Index. We show, using Light Detection and Ranging data to characterize deciduous and evergreen tree canopy, that the relationship between exposure to vegetation and childhood asthma onset is nonlinear and influenced by vegetation characteristics and season, from protective during the leaf-on season to harmful during the pollen season.

## Introduction

Asthma is the most common chronic disease in childhood,^1^ affecting an estimated 82 million children under the age of 15 worldwide in 2019.^2^ Its etiology is complex and multifactorial. Factors such as genetics, sex, breastfeeding, and indoor and outdoor air pollution have been related to asthma development in children,^1,3^ and growing evidence suggests that urban vegetation also influences childhood asthma development and symptoms. Yet, results from studies are mixed,^4,5^ with some suggesting a protective effect of vegetation cover,^6–11^ others an increased risk,^12–16^ or no effect at all.^8,16–18^ Most studies have used cross-sectional or ecological epidemiological designs, either based on surveys (e.g., parental reporting of a child’s symptoms in the past year)^8–11,15–17^ or health service utilization (e.g., emergency room visits and hospital admissions).^10,19^ Very few studies assessed the association between urban vegetation and the incidence of asthma development in children; most investigated the association with the prevalence of lifetime asthma.^6,8,12,14–17^ Furthermore, most studies assessed associations using the Normalized Difference Vegetation Index (NDVI).^6–8,11–13,16–18^ NDVI is a measure of total vegetation based on satellite images that cannot easily distinguish vegetation types, such as trees from shrubs and grass. A few studies used information on land use (e.g., crops, parks, forests, gardens; alone or in combination with NDVI).^6,15,16,20^ However, these also provide limited information because they do not distinguish between types of vegetation, such as trees and grass, or between types of trees.

Urban trees can potentially reduce air pollution and mitigate its effects on asthma development and exacerbation through mechanisms such as bioaccumulation and the deposition of gases and particulates on foliage.^4,21,22^ Yet, trees and other vegetation may also negatively impact air quality and increase asthma outcomes by releasing aeroallergens such as pollens and biogenic volatile organic compounds (BVOCs).^4,10,21,22^ Nonetheless, to our knowledge, only one study used tree canopy cover information (estimated from Light Detection and Ranging [LiDAR] data) to assess the association with asthma prevalence in children.^14^ Thus, it is unclear from published studies whether an increase in tree cover confers benefits or risks to childhood respiratory health.

Furthermore, the influence of trees on asthma development may vary by season. Pollen concentrations from trees, which may influence asthma,^23^ are only high in the Spring. Foliage, which may reduce air pollutant levels associated with asthma,^23^ is lost in the Fall. However, despite the complex interplay that may exist between trees and asthma outcomes, seasonal variations in vegetation (e.g., leaf-on and leaf-off seasons) and the potential for nonlinear associations have rarely been considered.

Using a longitudinal study design, we sought to examine the associations between residential urban total vegetation and tree cover and the development of childhood asthma (before age 13 years) in a birth cohort. NDVI, from satellite data, was used as a measure of total urban vegetation, while trees characterized based on LiDAR data were used to estimate evergreen and deciduous crown areas.

## Methods

### Study population

We used a birth cohort created with administrative health data that included all children born on the island of Montreal, Canada between 1 January 2000 and 31 December 2015. Age, sex, and residential six-digit postal codes throughout the follow-up were obtained from the administrative data. Children were followed from birth until they developed asthma, reached 13 years of age, moved out of the island of Montreal, were deceased, or until the end of the follow-up in 2015. The follow-up ended when children reached 13 years of age because most children reach puberty at this age, and hormonal changes that occur during this period are known to influence asthma.^24^ Incident cases of asthma were defined as children having at least two visits to a physician in a clinic or at the emergency room with a diagnosis of asthma within 24 months or at least one hospital separation with a diagnosis of asthma in any field; this definition was validated in children aged 0–18.^25^ Cases were deemed incident on the day of the year in which they met the case definition and could be considered incident only once. The birth cohort and asthma case definition are described in detail elsewhere.^26^

### Vegetation data

Tree crown area estimates were based on 2015 LiDAR point cloud data, as described in Zhao et al.^27^ LiDAR data was used to distinguish needle-leaf (evergreen) from broadleaf (deciduous) trees and to calculate the ground area covered by the crowns, expressed in square meters. NDVI data were obtained from the Canadian Urban Environmental Health Research Consortium (www.canue.ca) and used as an indicator of total vegetation; this data was produced based on Landsat 5 and 8 images at a resolution of 30 m.^28–33^ NDVI is a remotely sensed index of greenness ranging from −1 (water) to 1 (dense vegetation); the value for bare soil or concrete is 0.^34^ Very few negative NVDI values were observed; thus, no treatment of negative values was applied. We assigned 2015 growing season NDVI values (defined as 1 May to 31 August) and total tree canopy coverage on days of the pollen and leaf-on and -off seasons for a buffer of 250 m around each child’s residential postal code centroid throughout their follow-up. In urban settings, Quebec six-digit postal codes often represent a block face. When a change of address was recorded in the health database, the 2015 NDVI values and tree crown areas of the new residential postal code were assigned.

In Montreal, during the spring and summer months, most deciduous trees develop a green canopy (“leaf-on season”), while in the autumn and winter months, the leaves of deciduous trees are shed (“leaf-off season”). Evergreen trees keep their leaves on throughout the year. In this study, the leaf-on season was set from 1 May to 30 September for deciduous trees and over the entire year for evergreen trees. The pollen season was defined from 1 April to 30 June.^35^ Although trees are present all-year round, to reflect the absence of either pollen or leaves, the tree canopy for the pollen season was set to zero on the pollen-off days, while leaf-on exposures were set to zero during the leaf-off days. Thus, exposure is not life-long as it varies throughout the year, and with each move; a new line was added to the database when a change in exposure due to a move or a new season was noted (i.e., pollen- or leaf-on/off).

### Statistical analyses

We included in this study all children for whom complete information on vegetation exposure variables was available.

We used Cox proportional hazard models to evaluate the effect of time-varying exposure to urban vegetation on asthma development in our childhood birth cohort. We used age (i.e., time in days since birth) as the time axis to account for age-dependent changes in asthma development. Models were adjusted for sex, calendar year (to account for secular trends), and health and social services regions (to account for spatial clustering).^36^ Adjustment was done by considering specific baseline asthma hazard functions for each subgroup (i.e., stratification of the Cox model for sex, year, and health region).^36^ Montreal Island is divided into six health regions that coordinate local health and social services to their population, such as those from hospitals, medical clinics, and community organizations. Models were also adjusted for the time-varying indicator of material deprivation developed by Pampalon et al.^37^ This index, reported in quintiles with the first representing the least deprived, results from a principal component analysis regrouping indicators on instruction, employment, and revenue based on national census data^37^ (see supplementary material; http://links.lww.com/EE/A251).

Models assessing associations with asthma development included evergreen and deciduous trees’ crown areas during the leaf-on season (the whole year for evergreens) throughout the follow-up of each child. These models also included pollen season variables. However, due to the strong correlation between evergreen and deciduous trees’ crown areas during the pollen season (Pearson’s r = 0.73; *P* < 0.001), two distinct models were run: one for evergreen trees during leaf-on and pollen seasons, only adjusting for deciduous trees during the leaf-on season; and another with deciduous trees during pollen and leaf-on seasons, adjusting for evergreen leaf-on season (i.e., all-year). See Table S1; http://links.lww.com/EE/A251 for a list of all the models run and their adjustments.

For NDVI, we constructed similar models: one with a single exposure variable spanning the entire year, reflecting methods employed in previous studies;^6–9,12,13,16,18,38^ and a second with different pollen and leaf-on seasons NDVI values (Table S1; http://links.lww.com/EE/A251). For the later, NDVI values were only attributed on pollen days or during the leaf-on season; on all other days, the NDVI values were set to zero.

We conducted each of the above-described analyses using different sets of Cox models: (1) nonlinear models with a restricted cubic spline for vegetation exposure variables (knots = 3 or 4), and (2) models with vegetation variables categorized into three or four groups depending on the variable (Table S2; http://links.lww.com/EE/A251) and additional information in the supplementary material; http://links.lww.com/EE/A251. The Cox proportional hazard assumption was assessed through the visual inspection of risk through age per category of exposure. Trends with time were assessed with the Pearson product-moment correlation between the scaled Schoenfeld residuals and log(time) for each model covariate.^39^

Sensitivity analyses were performed to assess whether associations between vegetation and asthma development were independent of regional air pollutant levels (nitrogen dioxide and fine particles) (supplementary material; http://links.lww.com/EE/A251 for information on pollutant data used). In addition, the consistency of associations across different exposure buffers was assessed using buffers of 100, 500, and 1000 m around residential postal code centroids.

All computations were carried out using R Software 4.0.3 (http://cran-r-project.org); the packages used are listed in the the supplementary material; http://links.lww.com/EE/A251.

## Results

Of the 352,966 births recorded on the island of Montreal between 1 January 2000 and 31 December 2015, 20 participants had missing exposure information at birth and were excluded from the final analyses. We followed 352,946 children up to the end of age 12 years for a total of 1,732,064 person-years (Table 1). Among those, 30,816 children met the diagnostic criteria for asthma development for a mean rate of 17.8 new cases per 1,000 person-years and a mean follow-up of 4.9 ± 3.9 years. Asthma onset occurred more frequently at younger ages and in boys (60.79%). The onset rate peaked around age 2 years. Over the course of the follow-up, 492 children were excluded from our analysis as they were deceased, and another 80,023 (18% of the total included) moved outside of the island of Montreal (Table S3; http://links.lww.com/EE/A251). Moving patterns varied by quintiles of material deprivation, with children from the least deprived quintile (Q1: 23.4% of children who moved) almost twice as likely to move outside of the study area than those who belonged to the most deprived quintile (Q5: 12.3% of children who moved) (Table S4; http://links.lww.com/EE/A251).

**Table 1.** Description of the cohort born on the island of Montreal, Québec (Canada), 2000–2015, derived from the QCIDSS database

	n (% female)
Children born on the island of Montreal between 2000 and 2015	352,946 (50%)
Children with an asthma diagnosis	30,816 (39%)
Children censored	322,130 (50%)
Because of death	492 (43%)
Because of relocation	80,023 (49%)
Because of missing tree or vegetation exposure information at entry	20 (50%)
Because the study period ended	241,595 (50%)
Sex	Person-years of follow-up
Male	868,376.1
Female	863,688.4
Age group (years)	
<4	1,090,988.4
5–8	441,012.3
9–12	200,063.8
Material deprivation index (%)	
1 (least deprived)	309,095.2
2	306,802.1
3	316,322.3
4	340,565.1
5 (most deprived)	406,884.9
Missing index or invalid postal codes	52,394.9

**Figure 1.**
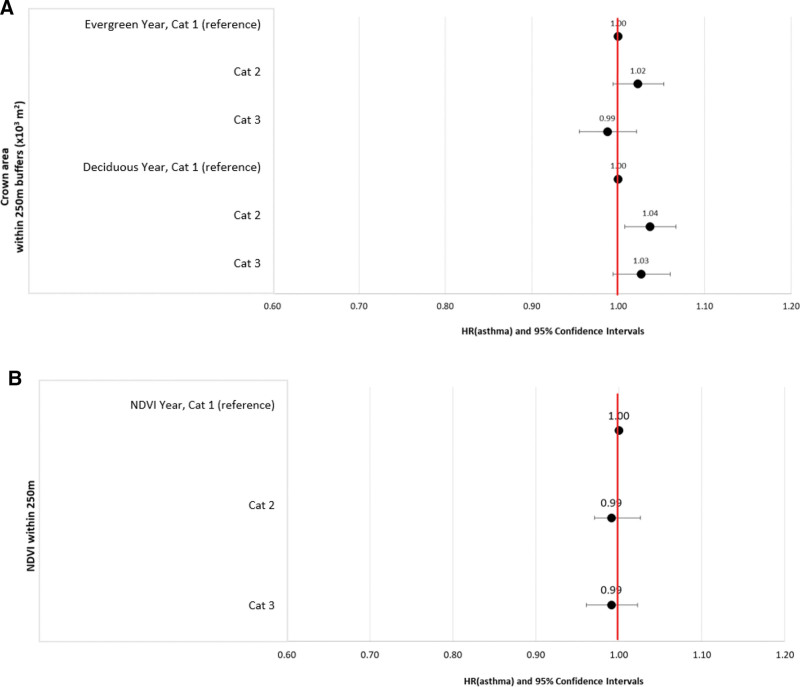
Hazard ratios and 95% confidence intervals for the adjusted associations between asthma development in children on Montreal Island and vegetation variables for the whole year: for evergreen and deciduous tree crown areas (A), and NDVI (B). The time axis is age in years since birth; models were adjusted for sex, calendar year, quintiles of material deprivation, and health and services regions. Categories for tree crown areas and NDVI estimates are for 250 m buffers around residential postal code centroids. Category (cat) limits (Table S2, http://links.lww.com/EE/A251) are as follows: Evergreen, year, Cat 1: [0.0, 2.2[10^3^ m^2^; Cat 2: [2.2, 4.5[10^3^ m^2^; Cat 3:]4.5, 53.2] 10^3^ m^2^ and deciduous year, Cat 1:]0.22, 22.4] 10^3^ m^2^; Cat 2:]22.4, 33.3] 10^3^ m^2^; Cat 3:]33.3, 168.3] 10^3^ m^2^. NDVI year Cat 1:]−0.02, 0.31], Cat 2:]0.31, 0.4] Cat 3:]0.40, 0.77]. Note that a deciduous tree crown area of 22.4 10^3^ m^2^ in a 250 m buffer area can be interpreted as follows: 11.4% of the ground surface within a 250 m buffer is covered by deciduous crown areas.

For vegetation exposure variables, the mean leaf-on crown areas within a 250 m buffer around residential postal codes were 4.1 and 1.7 times higher than during the pollen season for evergreen and deciduous trees, respectively (Table 2). Results were skewed due to the study design and construction of vegetation exposure estimates as seasonal estimates were set to zero on days outside of leaf-on and pollen seasons. Our findings suggest that relationships were nonlinear based on visual inspection of response curves (Figures S1–S3; http://links.lww.com/EE/A251) and comparison of akaike information criterion (AIC) for the different fitted response functions (Table S5; http://links.lww.com/EE/A251). Models with categorical all-year vegetation variables are shown in Figure 1 (data are presented in Tables S6–S9; http://links.lww.com/EE/A251). Comparing the second tercile of exposure distribution with the first for annual estimates of deciduous trees yielded higher hazard ratios (HR) of asthma development (HR = 1.04; 95% CI = 1.01, 1.07) (Table S6; http://links.lww.com/EE/A251). For all-year evergreen trees and NDVI, no association was observed.

**Table 2.** Distribution of estimated evergreen and deciduous trees’ crown areas and NDVI values within a 250 m buffer around the children’s residential postal codes throughout the follow-up (2000–2015). Values for categories of exposure variables are presented in Table S24

	Consideration of season^a^	Mean (±SD)	Min	25th	Median	75th	Max	IQR
Evergreens^b^ (×10^3^ m^2^)	All-year (leaf-on)	4.486 (4.278)	0	1.803	3.157	5.562	53.224	3.754
	Pollen	1.101 (2.864)	0	0	0	0	53.224	0
Deciduous^b^ (×10^3^ m^2^)	All-year	29.255 (13.740)	0.220	19.623	27.732	36.567	168.330	16.944
	Leaf-on	12.332 (16.981)	0	0	0	24.760	168. 300	24.760
	Pollen	7.196 (14.321)	0	0	0	0	168.330	0
NDVI^c^	Al year	0.365 (0.106)	-0.020	0.290	0.360	0.440	0.770	0.150
	Leaf-on	0.154 (0.193)	-0.020	0	0	0.330	0.770	0.330
	Pollen	0.089 (0.165)	-0.020	0	0	0	0.770	0

a Pollen and leaf-on season NDVI and crown area variables are present all-year round; values are set to the 2015 estimates for pollen and leaf-on season days and are set to zero on off-season days.

b Exposure derived from three-dimensional Airborne Laser Scanning (ALS) data (typical LiDAR system) mounted on an aerial vehicle, acquired in leaf-off conditions from 24 November to 8 December 2015, using a standard mono-spectral sensor (https://donnees.montreal.ca/ville-de-montreal/lidar-aerien-2015).

c Exposure estimates based on Landsat satellite images (30 m spatial resolution) for the growing season of 2015.

25th and 75th indicates first and third quartiles of distributions; IQR, interquartile range of exposure distributions; Min and Max, minimum and maximum observed exposure estimates (range); NDVI, Normalized Difference Vegetation Index; SD: standard deviation, based on exposure distributions within the cohort.

**Figure 2.**
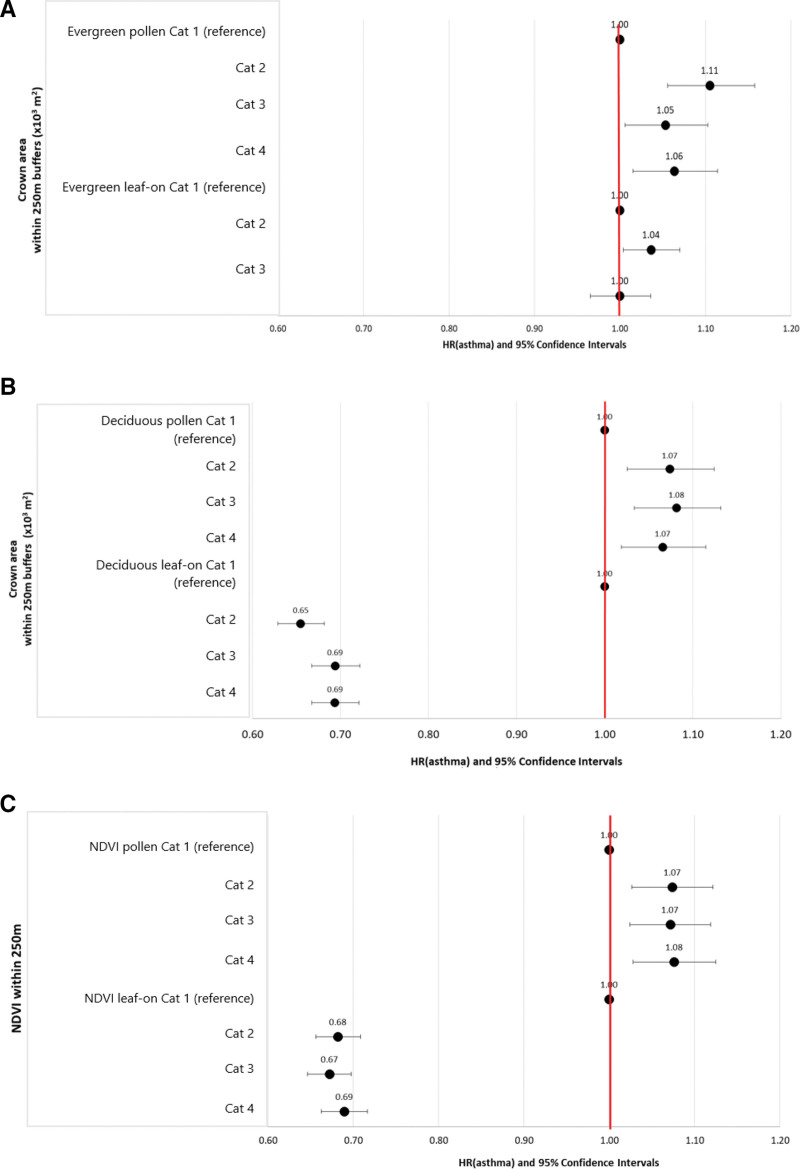
Hazard ratios and 95% confidence intervals for the adjusted associations between asthma development in children on Montreal Island and seasonal vegetation variables: for evergreen tree crown areas during pollen and leaf-on seasons (i.e., the whole year) adjusted for leaf-on deciduous tree crown areas (A), deciduous tree crown areas during pollen and leaf-on seasons adjusted for leaf-on evergreen tree crown areas (B), and NDVI estimates during pollen and leaf-on seasons (C). The time axis is age; models were adjusted for sex, calendar year, quintiles of material deprivation, and health and services regions. Categories for tree crown areas and NDVI estimates are for 250 m buffers around residential postal code centroids. Pollen and leaf-on tree and vegetation variables are obtained by setting to zero observations on pollen-off or leaf-off days. Category (cat) limits (Table S2, http://links.lww.com/EE/A251) are as follows: Evergreen pollen Cat 1 = 0, Cat 2 =] 0.03, 2.1], Cat 3 =] 2.1, 4.4], Cat 4 =]4.4. 53.2]. Evergreen, leaf-on (i.e., year), Cat 1: ]0.0, 2.2 [10^3^ m^2^; Cat 2:]2.2, 4.5[10^3^ m^2^; Cat 3:]4.5, 53.2] 10^3^ m^2^, Deciduous pollen Cat 1 = 0, Cat 2 =]0.22, 22.3], Cat 3 =]22.3, 33.1], Cat 4 =]33.1, 168.3], Deciduous leaf-on Cat 1 = 0, Cat 2 =]0.220, 22.3], Cat 3 =]22.3, 33.0], Cat 4 =]33.0, 168.3]. NDVI pollen Cat 1 = 0, Cat 2 = [−0.02, 0.31] excluding zeros, Cat 3 =]0.31, 0.40], Cat 4 =]0.40, 0.77], NDVI leaf-on Cat 1 = 0, Cat 2 = [−0.02, 0.31] excluding zeros, Cat 3 =]0.31, 0.40], Cat 4 =]0.40, 0.77].

On pollen season, adjusted HR nonlinearly increased with evergreen trees’ crown areas above zero (Figure 2A). Adjusted estimates of the association between deciduous trees’ crown areas on pollen and leaf-on seasons indicated opposing associations with asthma development. While during pollen season, higher HR of asthma development in children was observed, during the leaf-on season, deciduous trees’ crown areas were associated with lower HR (Figure 2B), indicating a protective effect. Similar patterns were observed for NDVI during pollen and leaf-on seasons (Figure 2C).

Sensitivity analyses presented in the supplementary material; http://links.lww.com/EE/A251 suggested that neither regional PM_2.5_ nor NO_2_ levels influenced the HR corresponding to each vegetation variable category (Table S10; http://links.lww.com/EE/A251). Analyses revealed similar results for all buffers (Tables S11 and S12; http://links.lww.com/EE/A251).

## Discussion

This study is the first to assess and compare associations between residential exposure to total urban vegetation and tree crowns, and asthma development in children. Through multivariable survival analysis, adjusting for confounding factors, we identified nonlinear associations between tree crown areas, NDVI, and childhood asthma development. These associations varied by tree type (evergreen and deciduous) and season (pollen, leaf-on, all-year). Seasonal patterns were particularly important for deciduous trees and NDVI. We found that both were associated with a reduced risk of asthma development during the leaf-on season, while during the pollen season, increased exposure to vegetation appeared to be associated with a higher HR of asthma development. In other words, asthma onset was more frequent during pollen season and less frequent during leaf-on season. The similarity among the seasonal patterns of the estimated associations between NDVI and deciduous trees suggests that the association between NDVI and childhood asthma development might be substantially attributable to the crowns of deciduous trees. Models with whole-year (leaf-on) evergreen tree crown areas yielded inconclusive results, while increased risks were observed during the pollen season.

Our results can be compared with previous studies that assessed the association between exposure to urban vegetation (and, for a few, urban trees) and childhood asthma. Among the few studies reporting on asthma development, inconsistent findings were described in published literature reviews.^4,5,40,41^ Our findings suggest that modeling flexibility (i.e., nonlinear modeling) and vegetation characteristics may account for the inconsistent results. Few studies have allowed for flexible modeling; those that did also reported nonlinear trends. In a birth cohort study by Markevych et al.,^42^ summer NDVI of residential addresses at birth were categorized into exposure terciles, and using spline modeling resulted in estimates that varied nonlinearly. With reference to the first tercile of NDVI, the second tercile was associated with an increased risk of asthma, while the third was associated with a decreased risk. Hsieh et al.^13^ in a case-control study based on medico-administrative data, also reported an increased risk of asthma development with higher levels of vegetation coverage (NDVI >0.4) until the highest levels, which appeared protective. Together with ours, these results suggest that associations vary with vegetation exposure levels and underline the importance of flexible modeling methods to ensure the precision and validity of results.

In addition to nonlinearity, our results support a common hypothesis whereby urban vegetation and urban trees negatively influence childhood asthma development over the pollen season. Surprisingly, we did not identify studies reporting associations with vegetation subdivided into seasonal or pollen exposures. Two studies^7,17^ did mention the use of seasonal NDVI values; neither reported seasonal estimates. In fact, Li et al.^17^ published results only for annual NDVI as estimates of association were unaffected by the use of seasonal NDVI values. Sbihi et al.^7^ used the annual average NDVI after observing a high correlation between NDVI levels measured across seasons. Most studies either used annual averages^8,42,43^ or assigned exposure based on maximum vegetation levels measured in spring or summer.^6,12,18,42–44^ Distinctive results might in part be attributable to the strategies that we employed for estimating seasonal exposure to vegetation (e.g., during pollen and leaf-on seasons).

Although we found that vegetation during the leaf-on season reduced the risk of childhood asthma onset, the mechanism by which this occurs is unresolved. Air pollution reduction is one main hypothesis. Although our analyses did not directly address this hypothesis, our sensitivity analyses showed a reduced risk of asthma development with increased vegetation in children, irrespective of the air pollutant levels. Another hypothesis is that the influence of vegetation on asthma is through stress reduction.

There are several strengths to our study, which uses secondary medico-administrative data to create a birth cohort and identify new asthma cases. Chiefly, our cohort covers virtually all children born on the island of Montreal between 2000 and 2015, as residents of Quebec benefit from universal access to health care, minimizing selection bias. Furthermore, the size of the cohort and longitudinal follow-up allowed for sufficient statistical power to detect small but important effects. Additionally, moving events within the study area were accounted for as the database provided updated residential postal codes throughout follow-up.

However, our study design and data sources have limitations as well. First, inherent to cohort studies using medico-administrative databases, was the limited inclusion of potentially important individual-level information about risk factors for asthma development that may vary with vegetation. Exposure to tobacco smoke, indoor air pollution, and allergens, as well as a family history of atopy, may have introduced residual confounding.^18^ The influence of some of these factors may have been at least partially accounted for by the adjustment for the ecological material deprivation index included in our models. Another limitation related to the use of medico-administrative databases to determine asthma onset relates to unequal access to care, which may vary with deprivation, although this may be minimal in Quebec due to universal health coverage.

The accuracy of asthma diagnoses recorded in medical and hospital discharge records is also a potential limitation of our study and could lead to the misclassification of new asthma cases. It can be challenging to distinguish asthma from other respiratory illnesses, such as bronchiolitis, in young children, and diagnoses are often based on family history and symptom patterns rather than objective testing. Nonetheless, the case definition employed is commonly used for surveillance in Canada and has been validated in children aged 0–18.^25^ The definition has a sensitivity and specificity of 89% and 72%, respectively, in children aged 0–17 years.^45^ Furthermore, previous studies using the same birth cohort to assess associations between environmental exposures and childhood asthma onset identified similar associations when limiting new cases of asthma detection to children aged four and older.^46^

As previously mentioned, due to data availability constraints, we assigned exposures for the entire follow-up period based on 1 year of data (2015 for NDVI and tree crown areas). Therefore, we could not account for changes in vegetation cover between years, which is another limitation. However, data for the greater region of Montreal suggests that vegetation coverage remained relatively stable between 2001 (76% vegetation coverage, NDVU >0.5) and 2011 (78%) and decreased slightly to 70% until 2019.^47^ We attribute changes in vegetation exposure based on the season or when a move occurred within the island of Montreal. Similarly, seasonal exposure estimates were constructed based on one-time measures and the same cutoff dates, although spring or pollen seasons may vary from year to year. Measurement bias would have been reduced using multiple measures of tree crown areas throughout the year, encompassing the different seasons, but these were, unfortunately, not available. In our study, changes in exposure estimates could solely be attributed to seasons or to moving within the island of Montreal. It appeared that those who moved outside the study area during follow-up were more likely to be less materially deprived, potentially inserting a selection bias as material deprivation was inversely associated with urban vegetation. Finally, exposure misclassification is also likely because daily mobility, which may become increasingly important as children age and go to school,^11^ was not considered.

## Conclusions

Our work suggests that urban vegetation, especially deciduous trees, affects children’s respiratory health. Results support that, although nonlinearly, deciduous tree crown areas are associated with reduced hazards of asthma development in children when leaves are present but increased hazards during the pollen season. This association was also reflected in models using NDVI, a measure of total vegetation cover, suggesting that most effects could be attributable to deciduous tree crowns. No associations were observed between NDVI or tree crown areas (for both deciduous and evergreen trees) and childhood asthma development when pollen and leaf-on seasons were not considered. These results support the importance of accounting for vegetation and tree characteristics (e.g., evergreen vs. deciduous) for children’s respiratory health and seasons. Future work will be needed to identify more specific vegetation characteristics that influence human health over a wide array of sites and environmental conditions to maximize benefits and minimize disservices of the green infrastructure. This information is instrumental in guiding urban greening initiatives to promote healthy, resilient cities.

## Conflict of interest statement

The authors declare that they have no conflicts of interest with regard to the content of this report.

## Acknowledgments

The authors wish to thank Julien Vachon (University of Montreal) for his assistance in producing some of the results. NDVI metrics, indexed to DMTI Spatial Inc. postal codes, were provided by CANUE (Canadian Urban Environmental Health Research Consortium).

## Supplementary Material
