# Supplementary material for: The influence of urban trees and total vegetation on asthma development in children
Source: Environ Epidemiol. 2023 Nov 16;7(6):e280. doi: 10.1097/EE9.0000000000000280 (PMC11189683; doi:10.1097/EE9.0000000000000280)
Supplement: Supplementary file 1 [file ee9-7-e280-s001.docx]

## Supplementary Material For “The influence of urban trees and total vegetation on asthma development in children” – Duquesne et al. 2023

### Supplementary methods

#### **Material deprivation**

The health administrative database does not contain individual-level socioeconomic status data. Accordingly, we used a specific area based ecological proxy for material deprivation developed by Pampalon et al.^1^ This index is reported in quintiles where the first quintile represents the least deprived. We attributed the value of the index of the dissemination area corresponding to the centroid of each postal code. A dissemination area includes on average 400 to 800 individuals ^2^ and thus, several postal codes may be included within the same dissemination area and have the same material deprivation quintile. The 2001 quintile data was used for the years 2000-2005, 2006 for the 2006-2010 years and 2011 for 2011-2015. Missing or invalid material deprivation values were classified into a 6th category assembling all missing or invalid entries for use in fully adjusted models.

**Table S1**: List of statistical models used to evaluate the association between childhood asthma onset and urban tree and vegetation variables. Each model was run twice, once using restricted cubic splines on exposure variables and once using categorized exposures.^a,b^

| Models | Exposure variables | Covariates |
| --- | --- | --- |
| Model 1 (a) | Evergreen crown area, all year + Deciduous crown area, all year | Sex^b^ + Year^b^ + RTS^b^ + Material Deprivation |
| Model 1 (b) | Evergreen crown area, all year + Evergreen crown area, Pollen^a^ + Deciduous crown area, leaf-on^a^ | Sex^b^ + Year^b^ + RTS^b^ + Material Deprivation |
| Model 1 (c) | Evergreen crown area, all year + Deciduous crown area, Pollen^a^ season + Deciduous crown area, leaf-on^a^ | Sex^b^ + Year^b^ + RTS^b^ + Material Deprivation |
| Model 2 (a) | NDVI, all year | Sex^b^ + Year^b^ + RTS^b^ + Material Deprivation |
| Model 2 (b) | NDVI leaf-on^a^ + NDVI pollen^a^ | Sex^b^ + Year^b^ + RTS^b^ + Material Deprivation |

*RTS = region of health services (n=6), Year = calendar year, leaf-on = leaf-on days over a year, pollen = pollen days over a year, NDVI = normalized difference vegetation index, Material deprivation= index of material deprivations, expressed as quintiles.*

*^a^Pollen and leaf-on season NDVI and crown area variables are present all year round; values are set to the 2015 estimates for pollen and leaf-on season days, and are set to zero on off-season days.*

*^b^ Adjustment with stratification of Cox models (i.e., assuming different baseline rates).*

#### **Vegetation and tree categories (Table S2)**

For whole year vegetation variables (i.e., leaf-on evergreen crown area and yearly NDVI and deciduous crown area), categories were based on terciles. For the remaining variables, due to the high number of null observations (on off-season days, values were set to zero), we grouped the null observations into one category and divided the remaining non-null observations into terciles, thus obtaining four categories for the pollen evergreen, pollen and leaf-on deciduous crown areas and pollen and leaf-on NDVI exposure variables.

**Table S2:** Category limits of tree and vegetation variables within 250 m buffers centered on children residential postal codes of the Montreal cohort.

| **Variables** | **Cat1** | **Cat 2** | **Cat 3** | **Cat 4** |
| --- | --- | --- | --- | --- |
| **Evergreen Crown Area (x10^3^ m^2^)** | | | | |
| **All year** | | | | |
| Category limits | [0; 2.2] | ] 2.2; 4.5] | ]4.5 ; 53.2] | - |
| Population (%) | 32.7 | 32.9 | 34.4 | - |
| **Pollen days** | | | | |
| Category limits | 0 | ] 0.03; 2.1] | ] 2.1; 4.4] | ]4.4; 53.2] |
| Population (%) | 75.4 | 8.1 | 8.1 | 8.4 |
| **Deciduous Crown Area (x10^3^ m^2^)** | | | | |
| **All year** | | | | |
| Category limits | [ 0.22; 22.4[ | [22.39, 33.3[ | [33.28,168.3] | - |
| Population (%) | 33.2 | 33.3 | 33.5 | - |
| **Leaf-on days** | | | | |
| Category limits | 0 | ]0.220; 22.3] | ]22.3; 33.0] | ]33.0;168.3 ] |
| Population (%) | 57.9 | 13.8 | 13.9 | 14.4 |
| **Pollen days** | | | | |
| Category limits | 0 | ]0.22; 22.3] | ]22.3; 33.1] | ] 33.1;168.3] |
| Population (%) | 75.4 | 8.1 | 8.1 | 8.4 |
| **NDVI** | | | | |
| **All Year** | | | | |
| Categories limits | ]-0.02;0.31] | ]0.32;0.4] | ]0.41;0.77] | - |
| Population (%) | 32.9 | 33.7 | 33.4 | - |
| **Leaf-on days** | | | | |
| Categories limits | 0 | ]-0.02;0[u ]0;0.31] | ]0.31;0.40] | ]0.40; 0.77] |
| Population (%) | 57.9 | 13.9 | 14.2 | 14.1 |
| **Pollen days** | | | | |
| Categories limits | 0 | ]-0.02;0[u ]0;0.31] | ]0.31;0.40] | ]0.40; 0.77] |
| Population (%) | 75.4 | 8.1 | 8.3 | 8.2 |

*Note: NDVI = Normalized difference vegetation index, Population(%) = proportion of person-years total exposed to these levels of tree or vegetation; cat= category.*

#### **Assessment of nonlinearity**

We produced several non-linear models with knots at different locations. We added a knot at crown area equal to zero. The other knots were placed at the category split or according to Harrell ^3^ which uses non-zero values. For all year NDVI and evergreen crown area variables, knots were placed according to Harrell ^3^ at the 10^th^, 50^th^, and 90^th^ quantile of the distribution.

We visually assessed the dose-response curves representing the associations between the continuous exposures to evergreen and deciduous crown areas and NDVI, and the risk of asthma development with the non-linear models with spline variables. We tested whether the goodness-of-fit of linear models was significantly improved upon the inclusion of the splined exposure variables. The comparison was performed based on the likelihood ratio test (LRT) with a cut-off p-value of 0.05. We also contrasted models with and without spline variables and categorized variables using the Akaike Information Criterion (AIC).

**Regional Air pollutants (PM_2.5_ and NO_2_)**

Ambient air pollution data included annual average concentration of PM ≤2.5*μ*m aerodynamic diameter (PM2.5) and annual nitrogen dioxide (NO2). Data were retrieved from the Canadian Urban Environmental Health Research Consortium (CANUE). Annual air pollution exposure levels were available through out the years of the study (from 2000 to 2015) and assigned to study participants based on updated six-digit residential postal codes.

Briefly, PM_2.5_ estimates were derived from 1 km aerosol optical depth satellite observations and the GEOS-Chem chemical transport model, and subsequently calibrated to regional ground-based observations a geographically weighted regression. PM_2.5_ metrics were indexed to DMTI Spatial Inc. postal codes. ^4,5^

Annual average NO_2_ concentrations estimates were obtained from a national land-use regression model that was based on measurements at Environment Canada’s National Air Pollution Surveillance (NAPS) system, satellite estimates of NO_2_, and geographic data such as road length, industrial land use, distance to highways and major roads. NO2 data were also indexed to DMTI Spatial Inc. postal codes. ^5–7^

#### **R packages used:**

For the Cox regression analyses, we used the following packages: “survival” v3.2-7,^8^ “Hmisc” v4.4-1,^9^ “survminer” v0.4.8,^10^ “wCorr” v1.9.1,^11^ “margins” v0.3.26,^12^ “ggeffects” v0.3.26, ^13^ “dplyr” v1.0.5,^14^ available via the CRAN project.

**Supplementary results**

**Table S3:** Characteristics of censored children by reason other than developing asthma or turning 13 from the Montreal Island birth cohort spanning from 2000 to 2015.

| **Reason** | **n (children)** | **Min (age)** | **Q1 (age)** | **Mean (age)** | **SD (age)** | **Median (age)** | **Q3 (age)** | **Max (age)** |
| --- | --- | --- | --- | --- | --- | --- | --- | --- |
| **Death** | 492 | 0 | 0 | 0.99 | 2.07 | 0 | 1 | 12 |
| **Moving out of the island of Montreal** | 80,023 | 0 | 1 | 2.78 | 2.61 | 2 | 4 | 12 |

*Note: Age is expressed in years since birth. n = number of children, Q1 = first quintile, Q3 = third quintile, SD = standard deviation, Min = minimum age observed, Max= maximal age observed*

**Table S4:** Quintiles of material deprivation of children who moved outside of the Island of Montreal and were censored from the birth-cohort which spanned from 2000 to 2015

| **Material deprivation (quintiles)** | **Frequency (children)** | **Proportion (%)** | **Cumulative frequency (children)** | **Cumulative percentage (%)** |
| --- | --- | --- | --- | --- |
| **Q1** | 18,063 | 23.38 | 18,063 | 23.38 |
| **Q2** | 20,266 | 26.23 | 38,329 | 49.61 |
| **Q3** | 16,409 | 21.24 | 54,738 | 70.85 |
| **Q4** | 13,039 | 16.88 | 67,777 | 87.72 |
| **Q5** | 9,486 | 12.28 | 77,263 | 100.00 |
| Frequency Missing = 2,760 | | | | |

| **S1A. S1B.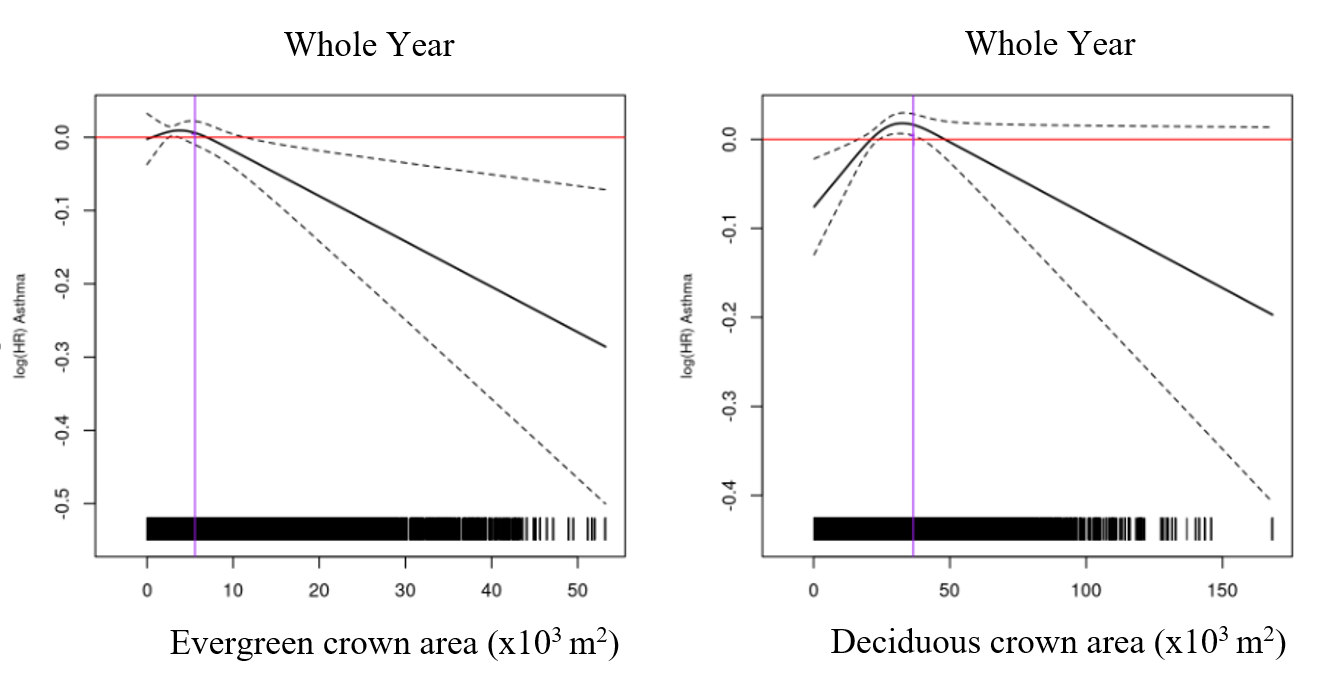** |
| --- |

***Figure S1****: Non-linear Cox proportional hazard functions using three knotted restricted cubic splines for hazard rates of childhood asthma by levels of whole year tree crown area for the Montreal Island birth-cohort spanning from 2000 to 2015. Time axis is age, models were adjusted for neighbourhood material deprivation (quintiles) and, were stratified for calendar year, sex, and regions of health and social services. We computed one model for evergreen and deciduous whole year crown area estimates. On the left (S1A) the hazard function for whole year evergreen crown area estimates adjusted for whole year deciduous crown area estimate. On the right (S1B) the hazard function for whole year deciduous crown area estimates adjusted for whole year evergreen crown area estimate. Restrictive cubic splines with three knots placed according to Harrel et al. (2001) (quantile 0.05, 0.5, 0.95) were used. Solid curves represent the mean log(HR) from the non-linear function fitted using restricted cubic splines. The dashed lines represent the 95% confidence interval distribution densities. Surrounding crown area re shown above the X-axis of each plot. Vertical lines represent the 75^th^ quantile of the distribution of each shown variable and the horizontal lines, log(HR) Asthma = 0.*

| **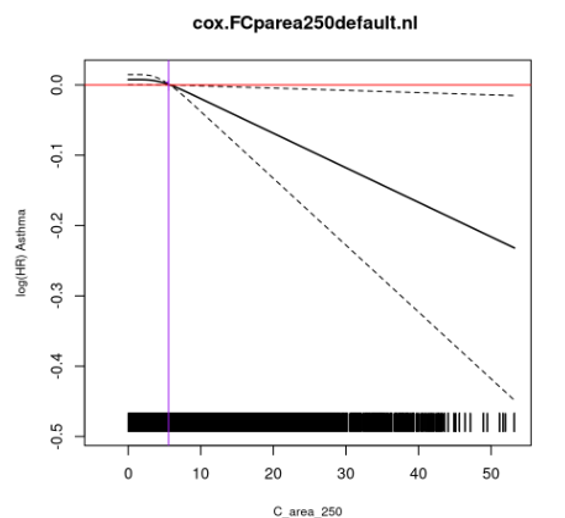S2A.**  Evergreen crown area (x10^3^ m^2^)  Leaf-on Season (Whole Year) | **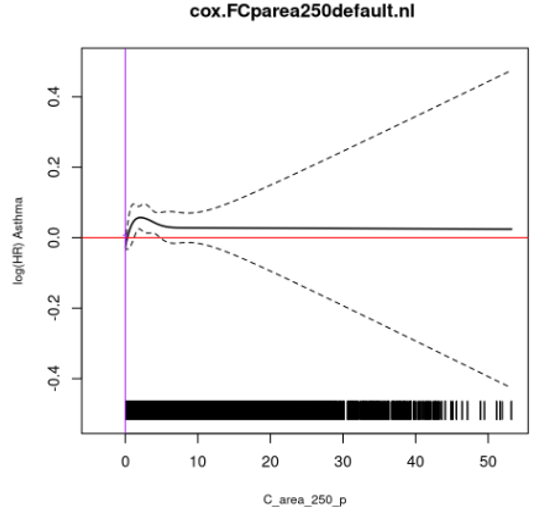S2B**  Pollen Season  Evergreen crown area (x10^3^ m^2^) |
| --- | --- |
| **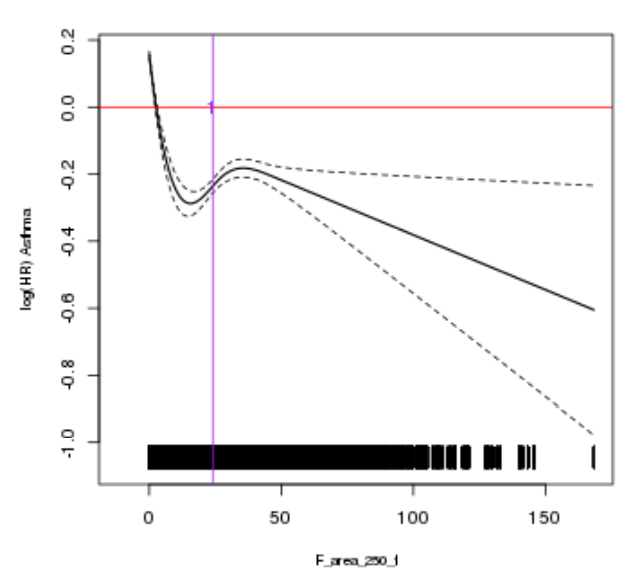S32C.**  Deciduous crown area (x10^3^ m^2^)  Leaf-on Season | **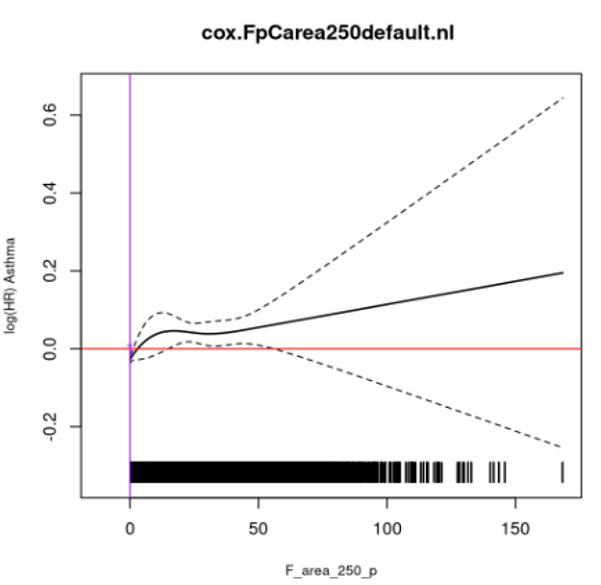 S2D.**  Deciduous crown area (x10^3^ m^2^)  Pollen Season |

***Figure S1****: Non-linear Cox proportional hazard functions using three knotted restricted cubic splines for hazard ratios of childhood asthma onset by levels of tree crown area for pollen and leaf-on seasons for the Montreal Island birth-cohort spanning from 2000 to 2015. Age was used as time scale, all models were adjusted for neighbourhood material deprivation (quintiles) and were stratified for calendar year, sex, and regions of health and social services. Two separate models were constructed. One for leaf-on (S2A) and pollen (S2B) evergreen crown area estimates adjusted for leaf-on deciduous crown area and, a second for deciduous leaf-on (S2C) and pollen days (S2D) estimates, adjusted for leaf-on evergreen crown area estimates. Restricted cubic splines with three knots, positioned according to the recommendations of Harrell (2001) (quantiles 0.05, 0.50 and 0.95), with a first knot at zero to account for null observations on leaf or pollen-off days. Specific response functions for each crown area variable are presented in separate figures with evergreen crown area in leaf-on (all year) (S2A) and pollen season (S2B); deciduous crown area in leaf-on season (S2C) and pollen season (S2D). Solid curves represent the mean log(HR) from the non-linear function fitted using restricted cubic splines. The dashed lines represent the 95% confidence interval distribution densities. Surrounding crown area re shown above the X-axis of each plot. Vertical lines represent the 75^th^ quantile of the distribution of each shown variable and the horizontal lines, log(HR) Asthma = 0.*

| 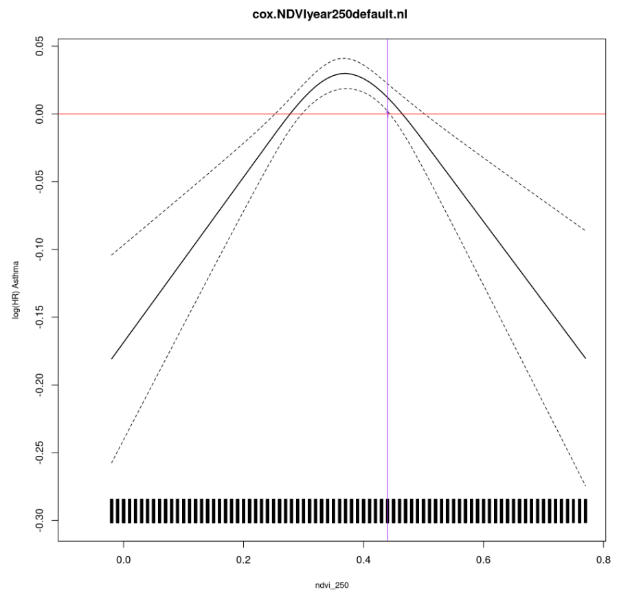**S3A.**  NDVI  Whole Year |  |
| --- | --- |
| 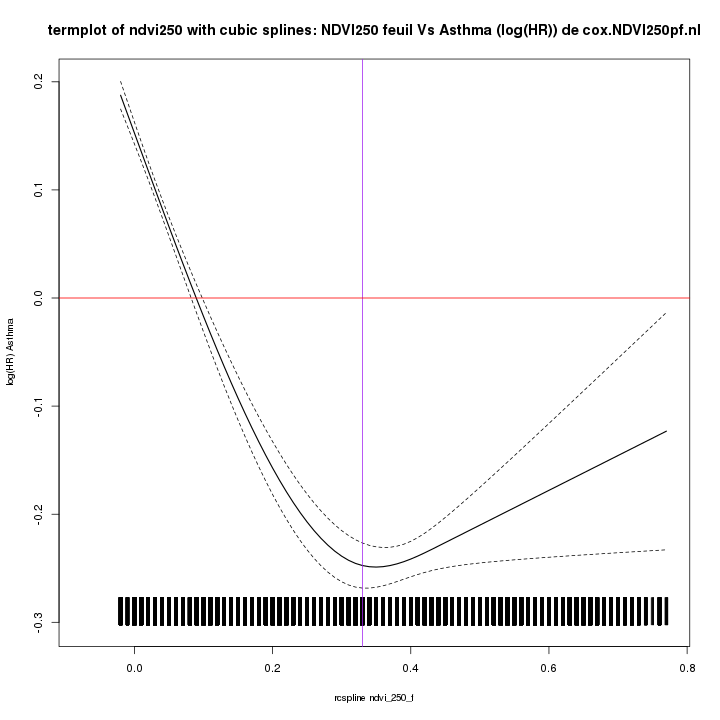**S3B.**  NDVI  Leaf-on Season | 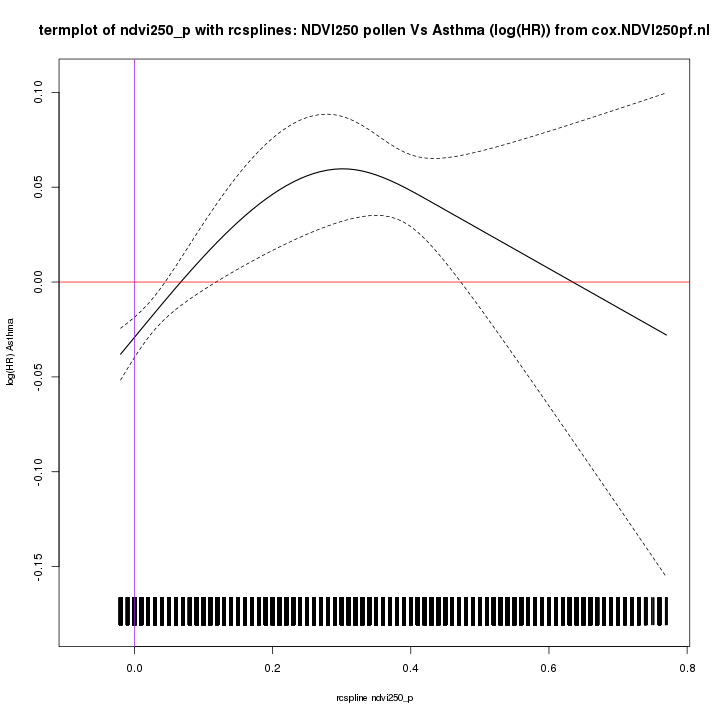  **S3C.**  NDVI  Pollen Season |

***Figure S3****: Non-linear Cox proportional hazard functions using three knotted restricted cubic splines for hazard rates of childhood asthma by levels of whole year, pollen and leaf-on NDVI for the Montreal Island birth-cohort spanning from 2000 to 2015. Time scale is age, models are adjusted for neighbourhood material deprivation (quintiles) and are stratified for calendar year, sex, and regions of health and social services. Two separate models are presented. Top figure (S3A) presents the hazard function for whole year NDVI values. In the bottom panel, on the left (S3B) the hazard function for leaf-on days NDVI adjusted for pollen days NDVI. On the right (S3C), the hazard function for pollen days NDVI adjusted for leaf-on days NDVI values. Restrictive cubic splines with three knots placed according to Harrel et al (2001) (quantile 0.05, 0.5, 0.95) were used. For pollen and leaf-on days a knot at zero was added to account for null observations on leaf or pollen-off days. Solid curves represent the mean log(HR) from the non-linear function fitted using restricted cubic splines. The dashed lines represent the 95% confidence interval distribution densities. Surrounding crown area re shown above the X-axis of each plot. Vertical lines represent the 75th quantile of the distribution of each shown variable and the horizontal lines, log(HR) Asthma = 0.*

**Table S5:** Model selection process based on AIC scores, searching for the most appropriate modeling approach to assess association between tree and vegetation variables within 250 m buffers centered on participants residential postal codes and asthma development in children.

|  | **Akaike information criterion (AIC)** | | | | |
| --- | --- | --- | --- | --- | --- |
| **Model**^a^ | Model 1 (a)  Whole Year deciduous + evergreen crown areas | Model 1 (b)  Pollen and leaf-on evergreen + leaf-on deciduous crown areas | Model 1 (c) Pollen and leaf-on deciduous + leaf-on evergreen crown areas | Model 2 (a)  NDVI whole year | Model 2 (b)  NDVI pollen and leaf-on |
| **Linear model** | 453,428 | 452,741 | 452,714 | 453,429 | 453,402 |
| **Non-linear model** | 453,418 (Fig S1) | 452,465 (Fig S2) | 452,463 (Fig S2) | 453,403 (Fig S3) | 453,378 (Fig S3) |
| **Categorized model** | 453,424 (Fig 1A) | 452,458 (Fig 2A) | 452,460 (Fig 2B) | 453,432 (Fig 1B) | 452,471 (Fig 2C) |

*Cox proportional hazards model with age as the timescale, adjusted for quintiles of material deprivation, sex, calendar year, and regional territory of social services. Nonlinear models were fitted using restricted cubic splines with three knots for variables without days set to zero due to season (ex. Evergreen, deciduous crown area and NDVI– whole year) and four knots for exposure variables with days set to zero (ex. Pollen and leaf-on season evergreen, deciduous and NDVI) (note that evergreen leaf-on season corresponds to evergreen whole year crown area since leaves remain present throughout the year). For three knots, knots were placed at the 10th, 50th and 95th percentiles of the exposure distribution. The four knots were placed at the 0th, 10th, 50th and 95th percentiles of the exposure distribution. NDVI: Normalized difference vegetation index*

^a^*Models are described in Table S1*

**Table S6***:* Hazard ratios and 95% confidence interval for the adjusted association between asthma development in children of the island of Montreal and categorized whole year tree crown area and vegetation variables estimates within 250 m buffers centered on children residential postal codes centroids. Data for Figure 1. ^a^

| **Term^b^** | | **HR** | **Lower 95%CI (HR)** | **Upper 95%CI (HR)** | |
| --- | --- | --- | --- | --- | --- |
| **Evergreen crown area – All year** | | | | | |
| Cat 1 (reference) | | 1 | 1 | 1 | |
| Cat 2 | | 1.0228 | 0.9940 | 1.0530 | |
| Cat 3 | | 0.9876 | 0.9549 | 1.0210 | |
| **Deciduous Crown Area –All year** | | | | | |
| Cat 1 (reference) | | 1 | 1 | 1 | |
| Cat 2 | | 1.0368 | 1.0076 | 1.0670 | |
| Cat 3 | | 1.0265 | 0.9940 | 1.0600 | |
| **NDVI – All year** | | | | | |
| Cat 1 (reference) | 1 | | 1 | | 1 |
| Cat 2 | 0.9913 | | 0.9822 | | 1.0141 |
| Cat 3 | 0.9913 | | 0.9774 | | 1.0054 |

*Note: Evergreen crown area values for the entire year correspond to leaf-on days, while all year deciduous crown areas include days where leaves area absent (leaf-off season). HR = Hazard Ratio ,95%CI = 95% Confidence Interval, NDVI = Normalized difference vegetation index, Cat = Category*

1. *All models are adjusted for quintiles of material deprivation (intercept = least deprived) and stratified for calendar year, sex, and regions of health and services. Age was used as time axis.*
2. *Categories limits for tree crown areas estimates are per 10^3^ m^2^; all estimates are computed within 250m buffers around residential postal code centroids (see Table S2).*

**Table S7:** Hazard ratios and 95% confidence interval for the adjusted association between asthma development in children of the island of Montreal and categorized evergreen crown areas for leaf-on and pollen season days, within 250 m buffers centered on residential postal code centroids. Data for Figure 2A.^a^

| **Term^b^** | **HR** | **Lower 95%CI (HR)** | **Upper 95%CI (HR)** |
| --- | --- | --- | --- |
| **Evergreen crown area – All year** | | | |
| Cat 1 reference) | 1 | 1 | 1 |
| Cat 2 | 1.0365 | 1.0040 | 1.0701 |
| Cat 3 | 0.9998 | 0.9651 | 1.0357 |
| **Evergreen Crown Area – Pollen days** | | | |
| Cat 1 (reference) | 1 | 1 | 1 |
| Cat 2 | 1.1053 | 1.0794 | 1.1318 |
| Cat 3 | 1.0531 | 1.0285 | 1.0783 |
| Cat 4 | 1.0635 | 1.0384 | 1.0892 |

*Note: HR = Hazard Ratio, 95%CI = 95% Confidence Interval, Cat = category*

1. *All models are adjusted for quintiles of material deprivation (intercept = least deprived) and stratified for calendar year, sex, and regions of health and services. Age was used as time axis.*
2. *Categories limits for tree crown areas estimates are per 10^3^ m^2^ within 250m buffers around residential postal code centroids (see table S2).*

**Table S8:** Hazard ratios and 95% confidence interval for the adjusted association between asthma development in children of the island of Montreal and categorized deciduous pollen and leaf-on crown areas within 250m buffers centered on residential postal code centroids. Data for Figure 2B.^a^

| **Term^b^** | **HR** | **Lower 95%CI (HR)** | **Upper 95%CI (HR)** |
| --- | --- | --- | --- |
| **Deciduous Crown Area – Leaf-on days** | | | |
| Cat 1 (reference) | 1 | 1 | 1 |
| Cat 2 | 0.655 | 0.629 | 0.682 |
| Cat 3 | 0.694 | 0.668 | 0.722 |
| Cat 4 | 0.694 | 0.668 | 0.7212 |
| **Deciduous Crown Area – Pollen days** | | | |
| Cat 1 (reference) | 1 | 1 | 1 |
| Cat 2 | 1.074 | 1.026 | 1.125 |
| Cat 3 | 1.081 | 1.034 | 1.132 |
| Cat 4 | 1.070 | 1.019 | 1.115 |

*Note: HR = Hazard Ratio, 95%CI = 95% Confidence Interval, Cat = category*

1. *All models are adjusted for quintiles of material deprivation (intercept = least deprived) and stratified for calendar year, sex, and regions of health and services. Age was used as time axis.*
2. *Categories limits for tree crown areas estimates are per 10^3^ m^2^ within 250m buffers around residential postal code centroids (see table S2).*

**Table S9:** Hazard ratios and 95% confidence interval for the adjusted association between asthma development in children of the island of Montreal and categorized pollen and leaf-on season NDVI estimates within 250m buffers centered on residential postal code centroids. Data for Figure 2C.^a^

| **Term^b^** | **HR** | **Lower 95%CI (HR)** | **Upper 95%CI (HR)** |
| --- | --- | --- | --- |
| **NDVI – Leaf-on days** | | | |
| Cat 1 (reference) | 1 | 1 | 1 |
| Cat 2 | 0.682 | 0.656 | 0.709 |
| Cat 3 | 0.672 | 0.647 | 0.699 |
| Cat 4 | 0.690 | 0.663 | 0.717 |
| **NDVI – Pollen days** | | | |
| Cat 1 (reference) | 1 | 1 | 1 |
| Cat 2 | 1.074 | 1.026 | 1.123 |
| Cat 3 | 1.071 | 1.024 | 1.121 |
| Cat 4 | 1.076 | 1.028 | 1.127 |

*Note: HR = Hazard Ratio, 95%CI = 95% Confidence Interval*

1. *All models are adjusted for quintiles of material deprivation (intercept = least deprived) and stratified for calendar year, sex, and regions of health and services. Age was used as time axis.*
2. *Categories limits for tree crown areas estimates are per 10^3^ m^2^ within 250m buffers around residential postal code centroids (see table S2).*

**Table S10** Cox regression results for risk of childhood asthma development in crude models and models adjusted for air pollution (NO_2_ and PM_2.5_)^a,b,c,d,e^

|  | Crude model | | | Model adjusted for air pollution | | |
| --- | --- | --- | --- | --- | --- | --- |
|  |  |  | PM_2.5_ | | NO_2_ | |
| Variables^f^ | HR | 95%CI | HR | IC 95% | HR | 95%CI |
| Model 1 | | | | | | |
| NDVI, year (cat 1) (Reference) | - | - | 1 | - | - | - |
| NDVI, year (cat 2) | 0.998 | 0.971-1.026 | 1.003 | 0.976-1.032 | 0.999 | 0.972-1.028 |
| NDVI, year (cat 3) | 0.992 | 0.961-1.023 | 1.008 | 0.977-1.041 | 0.996 | 0.964-1.029 |
| PM_2.5_ (per 1 μg/m^3^ increase) | - | - | 1.040 | 1.025-1.056 | - | - |
| NO_2_ (per 1 μg/m^3^ increase) | - | - | - | - | 1.001 | 0.998-1.004 |
| Model 2 | | | | | | |
| NDVI, leaf-on (cat 1) (Intercept) | 1 | - | 1 | - | 1 | - |
| NDVI, leaf-on (cat 2) | 0.682 | 0.656-0.710 | 0.679 | 0.653-0.706 | 0.681 | 0.655-0.709 |
| NDVI, leaf-on (cat 3) | 0.672 | 0.646-0.699 | 0.671 | 0.646-0.698 | 0.672 | 0.646-0.698 |
| NDVI, leaf-on (cat 4) | 0.690 | 0.663-0.717 | 0.694 | 0.667-0.722 | 0.691 | 0.664-0.719 |
| NDVI, pollen (cat 1) (Intercept) | 1 | - | 1 | - | 1 | - |
| NDVI, pollen (cat 2) | 1.073 | 1.025-1.122 | 1.069 | 1.022-1.119 | 1.072 | 1.024-1.121 |
| NDVI, pollen (cat 3) | 1.072 | 1.024-1.121 | 1.071 | 1.024 -1.120 | 1.071 | 1.024-1.120 |
| NDVI, pollen (cat 4) | 1.076 | 1.028-1.127 | 1.081 | 1.032-1.132 | 1.078 | 1.029-1.129 |
| PM2.5 (per 1 μg/m^3^ increase) | - | - | 1.040 | 1.026-1.056 | - |  |
| NO2 (per 1 μg/m^3^ increase) |  |  |  |  | 1.002 | 0.999-1.004 |
| Model 3 | | | | | | |
| Deciduous Crown Area, year (cat 1) (Intercept) | 1 | - | 1 | - | 1 | - |
| Deciduous Crown Area, year (cat 2) | 1.037 | 1.008-1.067 | 1.035 | 1.006-1.065 | 1.038 | 1.008-1.068 |
| Deciduous Crown Area, year (cat 3) | 1.027 | 0.995-1.061 | 1.023 | 0.990 -.056 | 1.028 | 0.995-1.062 |
| Evergreen Crown Area, year (cat 1) (Intercept) |  |  | 1 | - | 1 | - |
| Evergreen Crown Area, year (cat 2) | 1.024 | 0.995-1.053 | 1.027 | 0.998-1.057 | 1.025 | 0.996-1.055 |
| Evergreen Crown Area, year (cat 3) | 0.987 | 0.955-1.021 | 1.002 | 0.969-1.037 | 0.992 | 0.958-1.028 |
| PM_2.5_ (per 1 μg/m^3^ increase) | - | - | 1.039 | 1.024-1.054 | - | - |
| NO_2_ (per 1 μg/m^3^ increase) | - | - | - | - | 1.002 | 0.999-1.004 |
| Model 4 | | | | | | |
| Deciduous Crown Area, leaf-on (cat 1) (Intercept) | 1 |  | 1 | - | 1 | - |
| Deciduous Crown Area, leaf-on (cat 2) | 0.655 | 0.629-0.682 | 0.656 | 0.630-0.683 | 0.655 | 0.629-0.681 |
| Deciduous Crown Area, leaf-on (cat 3) | 0.694 | 0.668-0.722 | 0.695 | 0.668-0.722 | 0.695 | 0.668-0.722 |
| Deciduous Crown Area, leaf-on (cat 4) | 0.694 | 0.668-0.721 | 0.693 | 0.667-0.720 | 0.694 | 0.668-0.722 |
| Deciduous Crown Area, pollen (cat 1) (Intercept) | 1 | - | 1 | - | 1 | - |
| Deciduous Crown Area, pollen (cat 2) | 1.074 | 1.025-1.124 | 1.075 | 1.026-1.125 | 1.074 | 1.025-1.124 |
| Deciduous Crown Area, pollen (cat 3) | 1.081 | 1.033-1.132 | 1.081 | 1.033-1.132 | 1.081 | 1.033-1.132 |
| Deciduous Crown Area, pollen (cat 4) | 1.066 | 1.019-1.115 | 1.065 | 1.018-1.114 | 1.066 | 1.019-1.114 |
| Evergreen Crown Area, year (cat 1) (Intercept) | 1 | - | 1 | - | 1 | - |
| Evergreen Crown Area, year (cat 2) | 1.026 | 0.997-1.055 | 1.029 | 1.000-1.058 | 1.028 | 0.999-1.057 |
| Evergreen Crown Area, year (cat 3) | 0.991 | 0.960-1.024 | 1.005 | 0.973-1.038 | 0.996 | 0.963-1.031 |
| PM_2.5_ (per 1 μg/m^3^ increase) | - | - | 1.039 | 1.025-1.055 |  |  |
| NO_2_ (per 1 μg/m^3^ increase) | - | - | - | - | 1.002 | 0.999-1.004 |

*HR: Hazard Ratio; 95%CI :95% confidence interval around estimate.*

1. *Cohort cleaned for missing PM_2.5_ observations: N children = 352,946; mean follow-up = 1,792 days (or 4,9 years); total follow-up 1,733,250.84 person-year; N new asthma cases: 3,507 (60.6% male).*
2. *Cohort cleaned for missing NO_2_ observations: N children = 352,791; mean follow-up = 1,792 days (or 4,9 years); total follow-up= 1,731,946.307 person-years; N new asthma cases: 3,504 (60.6% male).*
3. *Distribution of PM_2.5_: mean annual concertation (Standard deviation (SD)) = 9.01 (SD: 1.12) µg/m^3^; median concentration (interquartile range (IQR)) = 8.9 (IQR: 0.7) µg/m^3^; range = 2.47 - 13.77 µg/m^3^.*
4. *Distribution of NO_2_: mean annual concertation (SD) = 13.31 (SD: 3.58) µg/m^3^; median concentration (IQR) = 13.89 (IQR: 5.09) µg/m^3^; range = 4.47 - 36.32 µg/m^3^*
5. *All models are adjusted for quintiles of material deprivation (intercept = least deprived) and stratified for calendar year, sex, and regions of health and services. Age was used as time axis.*
6. *Categories limits for tree crown areas estimates are per 10^3^ m^2^ within 250m buffers around residential postal code centroids (see table S2).*

**Table S11**: Cox regression results for risk of childhood asthma development for tree crown area for different buffer sizes around participants residential postal codes centroids for the Montreal birth cohort (2000-2015)^a^.

|  | **100m buffer** | | **250m buffer** | | **500m buffer** | | **1000m buffer** | |
| --- | --- | --- | --- | --- | --- | --- | --- | --- |
| **Variables^b^** | **HR** | **IC95%** | **HR** | **IC95%** | **HR** | **IC95%** | **HR** | **IC 95%** |
| **Model 1^c^** | | | | | | | | |
| **Deciduous year (cat 1) (reference)** | 1 | - | 1 | - | 1 | - | 1 | - |
| **Deciduous year Cat 2)** | 1.060 | 1.030-1.090 | 1.036 | 1.007-1.066 | 1.032 | 1.002-1.062 | 1.028 | 0.997-1.059 |
| **Deciduous year (Cat 3)** | 1.038 | 1.006-1.070 | 1.025 | 0.993-1.059 | 1.004 | 0.971-1.039 | 1.020 | 0.984-1.058 |
| **Evergreen year (Cat 1) (reference)** | 1 | - | 1 | - | 1 | - | 1 | - |
| **Evergreen year (Cat 2)** | 1.046 | 1.018-1.076 | 1.020 | 0.992-1.050 | 1.027 | 0.998-1.058 | 0.975 | 0.946-1.004 |
| **Evergreen year (Cat 3)** | 1.014 | 0.982-1.047 | 0.991 | 0.958-1.025 | 0.993 | 0.959-1.029 | 0.952 | 0.918-0.989 |
| **Model 2^c^** | | | | | | | | |
| **Deciduous, leaf-on (cat 1) (reference)** | 1 | - | 1 | - | 1 | - | 1 | - |
| **Deciduous , leaf-on (Cat 2)** | 0.663 | 0.638-0.690 | 0.656 | 0.630-0.683 | 0.656 | 0.630-0.683 | 0.659 | 0.633-0.686 |
| **Deciduous, leaf-on (Cat 3)** | 0.692 | 0.666-0.719 | 0.694 | 0.668-0.722 | 0.688 | 0.662-0.716 | 0.665 | 0.639-0.692 |
| **Deciduous , leaf-on (Cat 4)** | 0.687 | 0.661-0.714 | 0.693 | 0.666-0.720 | 0.699 | 0.673-0.727 | 0.720 | 0.692-0.748 |
| **Deciduous, pollen (cat 1) (reference)** | 1 | - | 1 | - | 1 | - | 1 | - |
| **Deciduous , pollen (Cat 2)** | 1.047 | 1.000-1.097 | 1.077 | 1.029-1.127 | 1.103 | 1.055-1.155 | 1.093 | 1.045-1.144 |
| **Deciduous, pollen (Cat 3)** | 1.102 | 1.053-1.152 | 1.080 | 1.032-1.130 | 1.071 | 1.023-1.121 | 1.094 | 1.045-1.146 |
| **Deciduous , pollen (Cat 4)** | 1.070 | 1.022-1.119 | 1.064 | 1.017-1.114 | 1.047 | 1.001-1.096 | 1.036 | 0.990-1.084 |
| **Evergreen, leaf-on (Cat 1) (reference)** | 1 | - | 1 | - | 1 | - | 1 | - |
| **Evergreen, leaf-on (Cat 2)** | 1.050 | 1.021-1.079 | 1.022 | 0.995-1.052 | 1.028 | 0.999-1.058 | 0.978 | 0.950-1.007 |
| **Evergreen, leaf-on (Cat 3)** | 1.022 | 0.991-1.054 | 0.995 | 0.963-1.028 | 0.993 | 0.961-1.027 | 0.957 | 0.924-0.991 |
| **Evergreen, leaf-on (Cat 4)** |  |  |  |  |  |  |  |  |

*HR: Hazard Ratio; 95% CI :95% confidence interval around estimate, Cat: category.*

1. *Cohort cleaned for missing vegetation exposure observations: N children = 352,946 (50.1% male); N asthma cases = 30,816 (60.6% male); N person-years of follow-up = 1,732,064*
2. *Category limits (population distribution (% total person-years)):*

*Deciduous Year 100m buffer: cat 1 = (0,30-.35] (33.2%) ; cat 2 = (3.35-5.53] (33.2%); cat 3 = (5.53-28.10] (33.6%) | 250m buffer : cat 1 = (0.22 – 22.40] (33.2%) ; cat2= (22.40-33.30] (33.3%) ; cat3 = (33.30-168.30] (33.5%)| 500m buffer: cat 1 = [5.3,90.7] (33.2%) ; cat2= (90.7,129] (33.3%) ; cat3 =(129,629] (33.5%) | 1000m buffer : cat 1 = [32.7,354] (33.2%) ; cat2= (354,487] (33.4%) ; cat3 = (487,1660] (33.4%)*

*Deciduous pollen 100m buffer : cat 1 = 0 (75.4%); cat 2 =(0 –3.35] (8.2%) ; cat 3 = (3.35-5.54] (8.2%) ; cat 4 =(5.54– 28.1] (8.2%) | 250m buffer : cat 1 = 0 (75.4%); cat 2 =(0–22.408] (8.2%) ; cat 3 = (22.41-33.31] (8.2%) ; cat 4 =(90.780 – 129.58] (8.2%) | 500m buffer : cat 1 = 0 (75.4%); cat 2 =(0 –354.52] (8.2%) ; cat 3 = (354.52-486.8] (8.2%) ; cat 4 =(486.8– 629] (8.2%) | 1000m buffer: cat 1 = 0 (75.4%) ; cat 2 =(0 –0.33] (8.2%); cat 3 = (0.33-0.40] (8.2%); cat 4 =(0.40 – 0,73] (8.2%)*

*Deciduous leaf-on 100m buffer : cat 1 = 0 (57.9%); cat2 = (0–3.34] (14.0%); cat 3 = (3.34-5.53] (14.0%) ; cat 4 =(5.53– 28.1] (14.2%) | 250m buffer : cat 1 = 0 (57.9%) ; cat 2 =(0–22.38] (14%); cat 3 = (22.38-33.26] (14%) ; cat 4 =(33.26 –] (14.2%) | 500m buffer : cat 1 = 0 (57.9%); cat 2 =(0 –90.71] (14.0%) ; cat 3 = (90.71-129.41] (14.0%) ; cat 4 =(129.41– 629] (14.2%) | 1000m buffer: cat 1 = 0 (57.9%); cat 2 =(0 –354.21] (14.0%); cat 3 = (354.21-486.63] (14.1%) ; cat 4 =(486.63– 1660] (14.1%)*

*Evergreen leaf-on (Year) 100m buffer : cat 1 = (0,0.292] (33.1%); cat 2 =(0.29-0.73] (33.2%) ; cat 3 = (5.53-28.10] (33.8%) | 250m buffer : cat 1 = (0.20-16 (33.0%); cat 2 = (2.16,4.53] (33.2%) ; cat 3 = (4.53,53.2] (33.8%) | 500m buffer : cat 1 = [0.468,9.31] (33.1%); cat 2 =(9.31,17.8] (33.2%) ; cat 3 = (17.8,155] (33.7%) | 1000m buffer: cat 1 = [2.28,39.2] (33.1%) ; cat 2 =(39.2,68.6] (33.2%); cat 3 = (68.6,443] (33.6%)*

*All models are adjusted for quintiles of material deprivation (intercept = least deprived) and stratified for calendar year, sex, and regions of health and services. Age was used as time axis.*

**Table S12**: Cox regression results for risk of childhood asthma development for NDVI for different buffer sizes around participants residential postal codes centroids for the Montreal birth cohort (2000-2015)^a^.

|  | **100m buffer** | | **250m buffer** | | **500m buffer** | | **1000m buffer** | |
| --- | --- | --- | --- | --- | --- | --- | --- | --- |
| **Variables^b^** | **HR** | **IC95%** | **HR** | **IC95%** | **HR** | **IC95%** | **HR** | **IC 95%** |
| **Model 1^3^** | | | | | | | | |
| **NDVI year (cat 1) (reference)** | 1 | - | 1 | - | 1 | - | 1 | - |
| **NDVI year (Cat 2)** | 1.034 | 0.975-1.031 | 0.998 | 0.971-1.026 | 0.993 | 0.966-1.021 | 0.993 | 0.966-1.021 |
| **NDVI year (Cat 3)** | 0.995 | 0.964-1.024 | 0.991 | 0.961-1.023 | 0.960 | 0.929-0.990 | 0.938 | 0.910-0.968 |
| **Model 2^c^** | | | | | | | | |
| **NDVI, leaf-on (cat 1) (reference)** | 1 | - | 1 | - | 1 | - | 1 | - |
| **NDVI , leaf-on (Cat 2)** | 0.668 | 0.642- 0.696 | 0.682 | 0.656-0.709 | 0.687 | 0.660-0.716 | 0.6809 | 0.656-0.707 |
| **NDVI, leaf-on (Cat 3)** | 0.690 | 0.664-0.717 | 0.672 | 0.647-0.699 | 0.668 | 0.643-0.695 | 0.678 | 0.651-0.707 |
| **NDVI , leaf-on (Cat 4)** | 0.683 | 0.657-0.710 | 0.690 | 0.663-0.717 | 0.688 | 0.662-0.715 | 0.683 | 0.657-0.711 |
| **NDVI, pollen (cat 1) (reference)** | 1 | - | 1 | - | 1 | - | 1 | - |
| **NDVI , pollen (Cat 2)** | 1.075 | 1.027-1.126 | 1.074 | 1.026-1.123 | 1.087 | 1.036-1.139 | 1.097 | 1.051-1.144 |
| **NDVI, pollen (Cat 3)** | 1.097 | 1.049-1.147 | 1.071 | 1.024-1.121 | 1.066 | 1.019-1.114 | 1.091 | 1.040-1.145 |
| **NDVI , pollen (Cat 4)** | 1.047 | 1.001-1.096 | 1.076 | 1.028-1.127 | 1.070 | 1.023-1.119 | 1.031 | 0.984-1.080 |

*HR: Hazard Ratio; 95% CI :95% confidence interval around estimate, Cat: category.*

1. *Cohort cleaned for missing vegetation exposure observations: N children = 352,946 (50.1% male); N asthma cases = 30,816 (60.6% male); N person-years of follow-up = 1,732,064*
2. *Category limits (population distribution (% total person-years)):*

*NDVI Year 100m buffer: cat 1 = (-0.09-0.31] (34.8%) ; cat 2 = (0.31-0.41] (32.7%); cat 3 = (0.41-0.79] (32.5%) | 250m buffer : cat 1 = (-0.02 – 0.32] (37.4%) ; cat2= (0.32-0.40] (29.2%) ; cat3 = (0.40-0.77] (33.4%)| 500m buffer: cat 1 = (0.0 – 0.32] (34.9%) ; cat2= (0.32-0.40] (32.9%) ; cat3 = (0.40-0.76] (32.2%) | 1000m buffer : cat 1 = (0 – 0.33] (37.2%) ; cat2= (0.33-0.40] (32.4%) ; cat3 = (0.40-0.73] (30.5%)*

*NDVI pollen 100m buffer : cat 1 = 0 (75.4%); cat 2 =(-0.0 –0.31] (7.6%) ; cat 3 = (0.31-0.40] (8.4%) ; cat 4 =(0.40 – 0,79] (8.5%) | 250m buffer : cat 1 = 0 (75.4%); cat 2 =(-0.02 –0.32] (8.1%) ; cat 3 = (0.32-0.40] (7.5%) ; cat 4 =(0.40 – 0,77] (9.0%) | 500m buffer : cat 1 = 0 (75.4%); cat 2 =(0 –0.32] (7.3%) ; cat 3 = (0.32-0.40] (8.5%) ; cat 4 =(0.40 – 0,76] (8.8%) | 1000m buffer: cat 1 = 0 (75.4%) ; cat 2 =(0 –0.33] (7.6%); cat 3 = (0.33-0.40] (8.7%); cat 4 =(0.40 – 0,73] (8.3%)*

*NDVI leaf-on 100m buffer : cat 1 = 0 (57.9%); cat2 = (-0.09 –0.31] (7.6%); cat 3 = (0.31-0.41] (8.4%) ; cat 4 =(0.41 – 0,79] (8.5%) | 250m buffer : cat 1 = 0 (57.9%) ; cat 2 =(-0.02 –0.32] (13.9%); cat 3 = (0.32-0.40] (12.8%) ; cat 4 =(0.40 – 0,77] (15.5%) | 500m buffer : cat 1 = 0 (57.9%); cat 2 =(0 –0.32] (12.5%) ; cat 3 = (0.32-0.40] (14.5%) ; cat 4 =(0.40 – 0,76] (15.1%) | 1000m buffer: cat 1 = 0(57.9%); cat 2 =(0 –0.33] (12.9%); cat 3 = (0.33-0.40] (14.9%) ; cat 4 =(0.40 – 0,73] (14.3%)*

1. *All models are adjusted for quintiles of material deprivation (intercept = least deprived) and stratified for calendar year, sex, and regions of health and services. Age was used as time axis.*

References

1. Pampalon R, Hamel D, Gamache P, Philibert MD, Raymond G, Simpson A. An Area-based Material and Social Deprivation Index for Public Health in Québec and Canada. *Can J Public Health Rev Can Santee Publique*. 2012;103:S17-S22.

2. MSSS gouv Qc. Informations Geographiques et de Population: Decoupage Territorial. Published online 2017. https://www.msss.gouv.qc.ca/professionnels/informations-geographiques-et-de-population/decoupage-territorial/

3. Harrell F. *Regression Modeling Strategies: With Applications to Linear Models, Logistic Regression, and Survival Analysis.* Springer; 2001. Accessed December 8, 2021. https://doi.org/10.1373/clinchem.2004.033688

4. Hammer MS, van Donkelaar A, Li C, et al. Global Estimates and Long-Term Trends of Fine Particulate Matter Concentrations (1998–2018). *Environ Sci Technol*. 2020;54(13):7879-7890. doi:10.1021/acs.est.0c01764

5. CanMap Postal Code Suite v2015.3. [computer file] Markham: DMTI Spatial Inc. Published online 2015.

6. Hystad P, Setton E, Cervantes A, et al. Creating National Air Pollution Models for Population Exposure Assessment in Canada. *Environ Health Perspect*. 2011;119(8):1123-1129. doi:10.1289/ehp.1002976

7. Weichenthal S, Pinault LL, Burnett RT. Impact of Oxidant Gases on the Relationship between Outdoor Fine Particulate Air Pollution and Nonaccidental, Cardiovascular, and Respiratory Mortality. *Sci Rep*. 2017;7(1):16401. doi:10.1038/s41598-017-16770-y

8. Therneau T. *A Package for Survival Analysis in R*.; 2021. https://CRAN.R-project.org/package=survival

9. Harrell F. *Hmisc: Harrell Miscellaneous*. CRAN; 2021. https://cran.r-project.org/package=Hmisc

10. Kassambara A, Kosinski M. *Survminer: Drawing Survival Curves Using “Ggplot2.”*; 2020. https://CRAN.R-project.org/package=survminer

11. Emad A, Bailey P. *WCorr: Weighted Correlations*.; 2017. https://CRAN.R-project.org/package=wCorr

12. Leeper T. *Margins: Marginal Effects for Model Objects*.; 2021.

13. Ludecke D. ggeffects: Tidy Data Frames of Marginal Effects from Regression Models. *J Open Source Softw*. 2018;3:772. doi:10.21105/joss.00772

14. Wickham H, Francois R, Henry L, Muller K. *Dplyr: A Grammar of Data Manipulation*.; 2021. https://CRAN.R-project.org/package=dplyr
